# Multilevel Factors Influencing Nurse–Patient Communication in Linguistically Diverse Healthcare Settings: A Qualitative Descriptive Study in Saudi Arabia

**DOI:** 10.3390/healthcare14142040

**Published:** 2026-07-08

**Authors:** Faihan F. Alshaibany, Abdullah M. Alharbi, Bader M. Almutairy, Majed M. Aljabri, Norah M. Alyahya, Bandar S. Alharbi, Waleed M. Alshehri, Abdulaziz M. Alodhailah, Thurayya Eid

**Affiliations:** 1 Department of Nursing Administration and Education, College of Nursing, King Saud University, Riyadh 11451, Saudi Arabia; 2 Community and Psychiatric Mental Health Nursing Department, College of Nursing, King Saud University, Riyadh 11451, Saudi Arabia; 3 Department of Medical-Surgical Nursing, College of Nursing, King Saud University, Riyadh 11451, Saudi Arabia

**Keywords:** nurse–patient communication, language barriers, cultural competence, ecological systems theory, Saudi Arabia, qualitative descriptive research, interpreter services, multilingual healthcare

## Abstract

**Background:** Effective nurse–patient communication is fundamental to quality care delivery, yet language barriers pose significant challenges in multicultural healthcare environments. In Saudi Arabia’s diverse healthcare landscape, nurses frequently encounter patients who do not speak Arabic, potentially compromising care quality and patient safety. **Objective:** To explore multilevel factors influencing communication between Saudi nurses and non-Arabic-speaking patients, using Bronfenbrenner’s ecological systems theory as a conceptual framework. **Design:** A qualitative descriptive study employing semi-structured interviews analyzed through reflexive thematic analysis. **Setting:** Four healthcare facilities (two governmental and two private hospitals) across Saudi Arabia. **Participants:** Eighteen Saudi registered nurses with experience caring for non-Arabic-speaking patients, recruited through purposive sampling. **Methods:** Semi-structured interviews (*n* = 18) were conducted in Arabic or English between November 2025 and February 2026. Data were analyzed using Braun and Clarke’s reflexive thematic analysis, organized within Bronfenbrenner’s ecological levels. Collaborative reflexive coding and member-checking with six participants supported analytical rigor. **Results:** Five main themes emerged: (1) Individual-level competencies and preparedness (microsystem), (2) Interpersonal dynamics and cultural sensitivity (microsystem), (3) Unit-level resources and organizational support (mesosystem), (4) Institutional policies and language services (exosystem), and (5) Healthcare system and societal influences (macrosystem). Participants identified language proficiency gaps, cultural misunderstandings, inadequate interpreter services, and systemic barriers as primary challenges affecting communication quality. **Conclusions:** Communication between Saudi nurses and non-Arabic-speaking patients is influenced by complex, interconnected factors across multiple ecological levels. Interventions should address individual competency development, organizational support systems, and policy-level changes to ensure equitable, safe, and effective communication for all patients.

## 1. Introduction

Effective communication between nurses and patients constitutes the cornerstone of therapeutic relationships and quality healthcare delivery [1,2]. In increasingly multicultural healthcare environments, language concordance between healthcare providers and patients has emerged as a critical determinant of care quality, patient safety, and health outcomes [3,4]. When patients and nurses do not share a common language, communication barriers can lead to misunderstandings, medication errors, reduced patient satisfaction, and compromised therapeutic relationships [4].

Saudi Arabia’s healthcare system serves a remarkably diverse patient population, including expatriate workers, pilgrims visiting for religious purposes, and international patients seeking medical care [5]. Saudi Arabia hosts over 13 million expatriate workers from various linguistic and cultural backgrounds, representing approximately 38% of the total population. Additionally, millions of Muslim pilgrims visit Saudi Arabia annually for Hajj and Umrah, many of whom require healthcare services during their stay. This linguistic diversity presents unique communication challenges for healthcare providers, particularly nurses who provide continuous patient care [6].

The recent literature has highlighted the multifaceted nature of language barriers in healthcare settings. Beyond simple linguistic differences, communication challenges encompass cultural variations in health beliefs, illness expressions, pain communication, and healthcare expectations [7]. These barriers can result in incomplete medical histories, inadequate patient education, reduced treatment adherence, and increased risk of adverse events [4].

The nursing profession’s emphasis on holistic, patient-centered care makes effective communication particularly crucial [8,9]. Nurses spend more time in direct patient contact than other healthcare professionals, requiring ongoing communication for assessment, education, emotional support, and care coordination. When language barriers impede this communication, the fundamental nursing processes of building rapport, conducting assessments, providing education, and ensuring patient safety become compromised [9].

### 1.1. Theoretical Framework

This study employs Bronfenbrenner’s ecological systems theory as its conceptual framework to understand the multilevel factors influencing nurse–patient communication [10]. This theory conceptualizes human development and behavior as influenced by nested environmental systems: microsystem (immediate environment), mesosystem (interactions between microsystems), exosystem (external environments indirectly affecting the individual), macrosystem (cultural context), and chronosystem (changes over time) [10].

Applied to nurse–patient communication, the microsystem encompasses individual nurse characteristics (language skills, cultural competence, communication abilities) and immediate patient interactions. The mesosystem involves relationships between different microsystems, such as interactions between nursing units, families, and interpreter services. The exosystem includes organizational policies, institutional resources, and healthcare system structures that indirectly influence communication. The macrosystem encompasses broader cultural, social, and policy contexts that shape healthcare delivery and communication expectations [11].

### 1.2. Literature Gap and Study Rationale

While international research has examined language barriers in healthcare, limited studies have specifically explored the experiences of Saudi nurses communicating with non-Arabic-speaking patients. Existing literature predominantly focuses on Western healthcare contexts, with minimal attention to Middle Eastern settings where linguistic diversity intersects with cultural and religious considerations [6,12].

Furthermore, most studies have employed quantitative approaches or focused on single-level factors, lacking systematic exploration of the multilevel influences on communication quality [12,13]. A systematic understanding of how individual, interpersonal, organizational, and systemic factors interact to influence communication outcomes is essential for developing effective interventions.

The significance of this research extends beyond Saudi Arabia’s borders, as healthcare systems worldwide grapple with increasing linguistic diversity. Understanding how nurses navigate communication challenges in a multicultural context can inform global nursing education, policy development, and practice improvement initiatives.

### 1.3. Aim and Research Questions

The aim of this study was to explore multilevel factors influencing communication between Saudi nurses and non-Arabic-speaking patients using Bronfenbrenner’s ecological systems theory as a guiding framework. The following research questions guided the inquiry: (1) What individual-level factors (microsystem) influence Saudi nurses’ communication with non-Arabic-speaking patients? (2) How do interpersonal and unit-level dynamics (mesosystem) affect communication quality? (3) What organizational and institutional factors (exosystem) facilitate or hinder effective communication? (4) How do broader healthcare systems and societal factors (macrosystem) influence nurse–patient communication? (5) What strategies do nurses employ to overcome communication barriers across different ecological levels?

## 2. Materials and Methods

### 2.1. Study Design

This qualitative descriptive study employed semi-structured interviews to explore participants’ experiences and perspectives on nurse–patient communication in multilingual healthcare settings. For the purposes of this study, key constructs are operationalized as follows. “Communication quality” refers to the degree to which nurses and patients achieve shared understanding and therapeutic exchange, encompassing linguistic accuracy, cultural appropriateness, and relational elements. “Cultural competence” denotes nurses’ ability to recognize, respect, and adapt to patients’ cultural health beliefs and communication norms. “Language proficiency” refers to self-reported ability to communicate in the patient’s preferred language. “Organizational support” encompasses institutional provision of interpreter services, translation resources, cultural competency training, and workload arrangements enabling adequate communication time. “Interpreter access” refers to the availability and practical usability of professional interpretation services, whether in-person, telephone-based, or technology-mediated. The qualitative descriptive design was selected for its ability to provide rich, detailed descriptions of complex phenomena while maintaining analytical flexibility, and is widely recognized as appropriate for exploratory nursing research where the goal is to describe and understand lived experience rather than generate theory [14,15].

### 2.2. Setting

The study was conducted across four healthcare facilities in Saudi Arabia: two governmental hospitals and two private healthcare institutions. These facilities were selected to represent diverse organizational contexts, patient populations, and resource availability. All participating facilities serve significant numbers of non-Arabic-speaking patients, including expatriate workers, international patients, and pilgrims from across the Muslim world.

### 2.3. Participants and Sampling

Participants were recruited through purposive sampling to ensure maximum variation in experience, educational background, and practice settings. Inclusion criteria were as follows: Saudi nationality; registered nurse status with active licensure; minimum of one year of experience in direct patient care; regular interaction with non-Arabic-speaking patients (at least weekly); and willingness to participate voluntarily. Nurses who had not worked directly with non-Arabic-speaking patients in the preceding six months were excluded.

The final sample comprised 18 Saudi nurses (*n* = 18) representing diverse backgrounds and experience levels. Sample size was guided by information power considerations [16]: recruitment continued until the data provided sufficient depth and breadth to address the research questions, as judged through discussion led by F.F.A. and A.M.A. [16]. Consistent with the interpretivist epistemology of reflexive thematic analysis, the research team assessed information sufficiency through ongoing discussion rather than applying a positivist saturation criterion. Recruitment continued until team consensus confirmed that the data richly addressed the research questions, and two additional interviews were conducted to confirm this judgment.

### 2.4. Data Collection

Data collection occurred between November 2025 and February 2026 following receipt of ethical approval. Semi-structured interviews were conducted using a comprehensive interview guide developed through literature review and expert consultation with specialists in nursing research, cross-cultural communication, and qualitative methodology. The interview guide (Supplementary File S2) covered six domains: demographic and professional background; general experiences with non-Arabic-speaking patients; factors influencing communication quality, including language barriers, cultural factors, interpreter and translation support, and administrative and environmental support; coping strategies and adaptations; impact on care delivery, including patient care quality, safety, adherence, and personal well-being; and support needs and improvement suggestions.

Interviews were conducted in participants’ preferred language (Arabic or English) by two bilingual members of the research team (F.F.A. and A.M.A.), both of whom hold doctoral qualifications and have substantive experience in qualitative nursing research and cross-cultural communication. A field note taker accompanied each interview session to document contextual observations and nonverbal cues. Conducting interviews in the participant’s dominant language is a methodologically sound practice that supports more authentic and nuanced data elicitation [17]. Each interview lasted approximately 45 to 60 min and was audio-recorded with participant consent. Verbatim transcription was completed by trained transcriptionists, with back-translation performed for Arabic-language interviews to ensure linguistic accuracy. Field notes were maintained throughout data collection to capture contextual information and researcher observations.

### 2.5. Ethical Considerations

The study was conducted in accordance with the principles of the Declaration of Helsinki. Ethical approval was obtained from the Institutional Review Board of King Saud University, Saudi Arabia (Reference: KSU-HE-25-1253, 4 November 2025). All participants provided written informed consent prior to data collection. Participation was voluntary, with the right to withdraw at any time without consequences. Data confidentiality and anonymity were maintained through de-identification procedures, including the assignment of participant codes (P1–P18), and secure encrypted data storage protocols.

### 2.6. Data Analysis

Data analysis followed Braun and Clarke’s six-phase reflexive thematic analysis approach [18,19]. All six phases proceeded inductively, with the research team remaining open to themes that cut across or did not fit the ecological levels. Once an initial thematic map had been constructed through inductive analysis, the research team subsequently examined whether and how the emergent themes corresponded to Bronfenbrenner’s ecological levels as an organizing structure for reporting. This approach treats the ecological framework as a post hoc organising lens rather than a deductive constraint on theme generation, in line with Braun and Clarke’s guidance on theoretically informed thematic analysis [18]. Reflexive thematic analysis was selected because it positions the researcher’s engagement with the data as central to the analytic process, supporting a theoretically flexible and interpretively rich account of the findings. The analytical process involved six phases: familiarization with data through repeated reading and initial noting; generating initial codes systematically across the entire dataset; searching for themes by collating codes into potential themes; reviewing themes to ensure coherence and distinctiveness; defining and naming themes; and producing the final analysis [19].

Two research team members coded the initial transcripts collaboratively, discussing and comparing their readings of the data on an ongoing basis. Rather than seeking consensus as a marker of coding reliability, these discussions served as a reflexive space in which differing interpretations were treated as an analytic resource that deepened engagement with the data, consistent with Braun and Clarke’s approach to reflexive thematic analysis. The coding framework was iteratively refined as analysis progressed, and themes were organized according to ecological system levels while maintaining openness to cross-cutting patterns and relationships between levels. Sustained reflexive dialogue among team members, documented in individual reflexivity journals, supported a theoretically informed and interpretively rich account of participants’ experiences. This study was reported in accordance with the Consolidated Criteria for Reporting Qualitative Research (COREQ) 32-item checklist (Supplementary File S1) to ensure comprehensive and transparent reporting of the qualitative research process.

### 2.7. Trustworthiness

Multiple strategies were employed to ensure research trustworthiness and rigor in accordance with the criteria proposed by Lincoln and Guba [20]. Credibility was enhanced through prolonged engagement with the data, member checking with six purposively selected participants (three from governmental and three from private facilities), who reviewed summary findings and interpretive accounts for accuracy and resonance, and investigator triangulation involving researchers with diverse professional and cultural backgrounds. Dependability was addressed through detailed audit trails and systematic documentation of analytical decisions at each phase. Confirmability was ensured through reflexive practices, including the maintenance of reflexivity journals by all team members, and peer debriefing sessions held at regular intervals throughout the study. Transferability was supported through rich, thick description of context, participants, and findings to allow readers to assess the applicability of findings to other settings.

### 2.8. Reflexivity Statement

The research team comprised nursing academics and clinicians from King Saud University with professional experience in Saudi healthcare settings, nursing education, and qualitative research methodology. The lead interviewers (F.F.A. and A.M.A.) hold senior academic positions and have personal familiarity with clinical contexts in Saudi hospitals. This insider positioning facilitated participant rapport but carried the risk of confirmatory interpretation. To manage this, all team members maintained reflexivity journals documenting assumptions, analytical decisions, and interpretive tensions. Peer debriefing sessions held at three-week intervals throughout the study interrogated emerging interpretations and ensured findings reflected participants’ meanings rather than researchers’ prior frameworks.

## 3. Results

### 3.1. Participant Characteristics

The 18 participants ranged in age from 25 to 45 years, with nursing experience spanning 2 to 20 years. All participants reported weekly encounters with non-Arabic-speaking patients, with frequency ranging from 2 to 3 patients per week to daily interactions. The most commonly encountered patient languages included English, Urdu, Bengali, Filipino languages, and various African languages. The majority held a bachelor’s degree (55.6%) and worked in government hospitals (55.6%). More than half (61.1%) received some form of cultural competency training, though scope and depth varied considerably across institutions. The most commonly encountered non-Arabic languages were English, Urdu, Bengali, Filipino languages (Tagalog/Cebuano), and various sub-Saharan African languages (Table 1).

### 3.2. Thematic Analysis Results

Five main themes emerged from the data, organized according to ecological system levels. These themes demonstrate the complex interplay of factors across multiple levels that influence communication quality between Saudi nurses and non-Arabic-speaking patients. Each theme is described below with illustrative participant quotations.

#### 3.2.1. Theme 1: Individual-Level Competencies and Preparedness (Microsystem)

Participants consistently identified personal factors that influenced their communication effectiveness with non-Arabic-speaking patients. Language proficiency emerged as a fundamental competency, with nurses expressing varying levels of confidence in their English and other language skills. Beyond linguistic abilities, participants described the importance of cultural awareness and sensitivity in effective communication, with nurses who had previous intercultural exposure or formal training demonstrating greater confidence in navigating cultural differences.

“*My English is okay for basic conversation, but when it comes to explaining complex medical procedures or pain assessment, I sometimes struggle to find the right words. I worry that the patient does not fully understand what I am trying to explain*.”

(Participant 7)

Communication skills and adaptability were identified as crucial individual competencies. Participants described developing various strategies to enhance understanding, including simplified language use, nonverbal communication, and creative problem-solving approaches. The importance of attitude was equally evident, as nurses who viewed linguistic diversity as an opportunity for growth reported more positive experiences and better patient outcomes than those who perceived it as a burden.

“*Working with patients from different cultures has taught me that communication is not just about language. Sometimes a patient’s silence does not mean they understand or agree; it might be respect or cultural politeness. I have learned to ask more questions and check understanding differently*.”

(Participant 3)

Self-efficacy beliefs played a crucial role in communication attempts. Nurses with higher confidence in their communication abilities were more likely to engage in extended patient interactions and employ diverse communication strategies, suggesting that targeted confidence-building interventions may be as important as language instruction.

“*At first, I was intimidated by patients who could not speak Arabic. But now I see it as a challenge that makes me a better nurse. Each interaction teaches me something new about communication and patience*.”

(Participant 15)

#### 3.2.2. Theme 2: Interpersonal Dynamics and Cultural Sensitivity (Microsystem)

The quality of nurse–patient relationships was significantly influenced by cultural understanding and mutual respect. Participants described how cultural differences in health beliefs, family involvement, gender considerations, and communication styles affected their interactions. These interpersonal dimensions operated at the microsystem level and were often as consequential as language proficiency in determining communication success. Notably, nurses with strong cultural understanding but limited language proficiency frequently achieved better communication outcomes than those with advanced language skills but limited cultural insight.

“*Some patients come from cultures where family members make healthcare decisions, not the patient directly. I had to learn to include family members in discussions and respect these dynamics, even though it is different from our usual approach*.”

(Participant 11)

Gender considerations emerged as particularly important in the Saudi healthcare context, where cultural and religious norms influence patient–provider interactions. Female nurses described challenges when caring for male patients from certain cultural backgrounds, while also noting instances where shared gender facilitated communication and the establishment of trust.

“*As a female nurse, some male patients from conservative cultures initially seemed uncomfortable. But once I showed respect for their cultural boundaries and involved their families appropriately, communication improved significantly*.”

(Participant 4)

Nonverbal communication patterns varied significantly across cultures, leading to potential misunderstandings. Participants learned to recognize and adapt to different cultural expressions of pain, distress, and satisfaction. Religious and cultural practices, including prayer times, dietary restrictions, and modesty requirements, also influenced communication patterns and required nurses to develop culturally responsive approaches.

“*I noticed that some patients smile and nod even when they do not understand or are in pain. In their culture, this might be politeness, but I learned to look for other signs and ask more specific questions to really assess how they are feeling*.”

(Participant 15)

#### 3.2.3. Theme 3: Unit-Level Resources and Organizational Support (Mesosystem)

Workplace dynamics and available resources significantly influenced communication quality. Units with supportive cultures that valued diversity and patient-centered care facilitated better communication outcomes. The mesosystem-level factors described by participants were frequently shaped by the degree to which unit leadership actively modeled and promoted inclusive communication practices.

Colleague support emerged as a crucial mesosystem factor. Nurses described how multilingual colleagues, charge nurses, and physicians served as informal interpreters and cultural mediators, often filling the gap left by the absence of formal language support services. However, this reliance on informal peer assistance also created challenges when multilingual staff were unavailable or were occupied with their own responsibilities.

“*We have a nurse from the Philippines who speaks Tagalog, and another who is fluent in Urdu. When we have patients who speak these languages, we help each other. It is not official, but it works better than waiting for an interpreter*.”

(Participant 2)

Workload and time constraints posed significant challenges to effective communication. Participants described feeling rushed during patient interactions, limiting their ability to ensure understanding and address concerns adequately. This pressure was particularly acute during handovers, procedures, and complex clinical situations where communication accuracy was most critical.

“*Communication takes more time when there is a language barrier. You need to speak slowly, repeat things, check understanding. But when you have six patients and medications due, it is difficult to spend the time you know is needed*.”

(Participant 9)

Unit leadership attitudes toward cultural diversity significantly influenced the overall communication climate. Supportive nurse managers who encouraged cultural competence and provided resources facilitated better patient interactions, whereas units where diversity was treated as a secondary concern tended to have less consistent and less effective communication practices.

“*Our nurse manager always tells us that good communication is part of quality care, regardless of language barriers. She gives us extra time when needed and encourages us to use all available resources*.”

(Participant 12)

#### 3.2.4. Theme 4: Institutional Policies and Language Services (Exosystem)

Organizational policies and available language support services significantly influenced communication effectiveness. Participants reported wide variation in institutional support across different facilities, reflecting inconsistent implementation of language access policies and uneven investment in supportive infrastructure.

Interpreter services, where available, received mixed reviews. While participants appreciated professional interpretation, they noted challenges related to availability, timeliness, and quality of service. The gap between having a policy that endorsed interpreter use and making such services practically accessible was a recurring source of frustration.

“*We have telephone interpreters, but sometimes the connection is poor or the interpreter does not understand medical terminology. Face-to-face would be better, but it is not always available, especially during night shifts*.”

(Participant 14)

Technology resources, including translation applications and multilingual patient education materials, were inconsistently available across institutions. Some facilities provided tablets with translation applications, while others relied on nurses’ personal devices or makeshift solutions. Participants consistently noted that while digital tools supported basic communication, they were insufficient for complex clinical discussions requiring nuanced and accurate interpretation.

Staff training and education policies varied significantly. While some institutions provided cultural competency training, others offered limited or no structured preparation for communicating with diverse patient populations. Documentation and communication protocols also lacked specific guidance for addressing language barriers, leaving nurses to develop their own approaches in the absence of institutional direction.

“*There is no clear policy about what to do when we cannot communicate with a patient. Do we wait for an interpreter? Use family members? Use apps? Each nurse does something different*.”

(Participant 6)

#### 3.2.5. Theme 5: Healthcare System and Societal Influences (Macrosystem)

Broader healthcare system policies and societal attitudes influenced communication dynamics. Participants described how national healthcare policies, professional regulations, and cultural contexts shaped their day-to-day communication experiences, often in ways that were subtle yet pervasive.

The Saudi healthcare system’s emphasis on providing care to diverse populations, including pilgrims and expatriate workers, created both opportunities and challenges. While institutional missions embraced diversity and positioned linguistic inclusivity as a component of service excellence, practical implementation often lagged behind policy intentions, leaving frontline nurses to bridge the gap through individual effort and informal peer support.

“*Saudi Arabia welcomes millions of pilgrims every year, and we are supposed to provide excellent care regardless of language. But the reality is that communication barriers make this challenging. There should be clearer standards about language support and cultural competency requirements*.”

(Participant 1)

Professional nursing standards and regulations provided some guidance for communication practices, but participants noted gaps in specific requirements for linguistic competency or cultural responsiveness. Societal attitudes toward linguistic diversity also influenced both patient expectations and nurse behaviors, with participants describing varying levels of community and institutional support for multilingual healthcare services. Healthcare financing and resource allocation policies further affected the availability of language support, as budget constraints often limited institutional investment in interpretation services and cultural competency training.

### 3.3. Cross-Cutting Patterns and Relationships

Several patterns emerged across ecological levels, highlighting the interconnected nature of factors influencing communication. Individual competencies interacted with organizational support to determine communication effectiveness. Nurses with strong language skills but limited institutional support reported frustration and suboptimal outcomes, while those with moderate skills but robust organizational backing achieved better results, underscoring the importance of a systemic, rather than purely individualistic, approach to communication improvement [21,22].

Time pressures generated cascading effects across levels, from increased individual stress and diminished communication quality to unit-level tensions and broader institutional quality concerns. Conversely, strengths at one level could mitigate limitations at others; for example, strong peer support partially compensated for limited formal interpreter services. Notably, the interaction between cultural competence and language skills was particularly influential [23]: nurses with high cultural awareness but limited language proficiency often achieved better communication outcomes than those with strong language skills but limited cultural understanding. This finding suggests that cultural education may yield proportionally greater returns than language training alone in certain clinical contexts.

## 4. Discussion

### 4.1. Principal Findings

This study reveals that communication between Saudi nurses and non-Arabic-speaking patients is influenced by complex, interconnected factors across multiple ecological levels. The findings demonstrate that effective communication requires more than individual language proficiency, encompassing cultural competence, organizational support, adequate resources, and systemic policy alignment. These multilevel interdependencies have important implications for how interventions are designed and evaluated [21].

At the microsystem level, individual nurse characteristics significantly influenced communication quality. Language proficiency emerged as a foundational but insufficient competency, requiring supplementation with cultural awareness, adaptability, and patient-centered attitudes. These findings align with international research emphasizing the multidimensional nature of cross-cultural communication competence [9]. The importance of cultural sensitivity extends beyond language translation to encompass understanding diverse health beliefs, communication styles, and care expectations, resonating with transcultural nursing theory and research emphasizing the need for culturally congruent care [24,25].

At the mesosystem level, collegial support and unit culture emerged as crucial facilitators of effective communication. The finding that nurses rely heavily on multilingual colleagues for informal interpretation highlights both resourcefulness and potential risks, as untrained interpreters may lack medical terminology knowledge or confidentiality training [26,27]. Exosystem factors, particularly organizational policies and language services, significantly influenced communication outcomes. The variation in institutional support across different facilities suggests inconsistent implementation of evidence-based language access practices, aligning with literature documenting wide disparities in language service provision across healthcare institutions [28,29].

Macrosystem influences, including healthcare policies and societal attitudes, created the broader context within which communication occurred. The finding that policy intentions often exceeded practical implementation resonates with implementation science literature highlighting the complexity of translating policy into practice [30,31].

### 4.2. Novel Contributions

This study makes three distinct contributions to nursing literature. First, the application of Bronfenbrenner’s ecological theory to nurse–patient communication in a Middle Eastern context is, to our knowledge, without precedent in the published literature, though multilevel and social-ecological framings of language-access strategies for hospital patients have recently gained traction internationally [32]. Second, the identification of the cultural competence–language proficiency interaction (where cultural awareness partially substitutes for language skill) offers a mechanistic insight with direct curriculum implications. Third, the documentation of informal peer interpretation as a system-level substitution strategy reframes this practice as a target for policy intervention rather than individual correction. The identification of specific cultural considerations, such as gender dynamics and family decision-making patterns, adds to the literature on culturally responsive nursing care in Middle Eastern settings [6,33,34,35].

The finding that informal colleague support networks often compensate for inadequate formal language services highlights the importance of peer relationships in multicultural care environments, with implications for staff scheduling, team composition, and professional development strategies. The study also reveals the critical role of time pressures in creating cascading effects across ecological levels, suggesting that workload management is essential for effective cross-cultural communication [36,37].

### 4.3. Implications for Nursing Practice

The multilevel nature of communication challenges requires comprehensive, coordinated interventions addressing individual, unit, organizational, and system factors simultaneously. Single-level interventions are unlikely to achieve sustainable improvements in communication quality. Individual nurses should pursue ongoing professional development in language skills and cultural competence, while recognizing that personal efforts alone cannot overcome systemic barriers [15,35,38]. Peer support networks and mentoring relationships can facilitate knowledge sharing and skill development. Nurse managers should foster unit cultures that value diversity, provide adequate time for patient communication, and facilitate access to language support resources, with clear protocols for accessing interpreter services and utilizing multilingual staff safely and appropriately. Healthcare organizations should invest in comprehensive language access infrastructure, including professional interpreter services, translation technology, and multilingual educational materials, with regular assessment of language service quality and accessibility [37].

### 4.4. Implications for Nursing Education

Nursing education programs should integrate comprehensive cultural competency and communication skills training throughout curricula rather than relegating these topics to isolated courses [9]. Simulation-based learning experiences can provide safe environments for practicing cross-cultural communication skills, while language learning opportunities, particularly in healthcare-specific English and other commonly encountered languages, should be available for both nursing students and practicing nurses. Interprofessional education experiences involving interpreters, social workers, and cultural liaisons can help nurses understand collaborative approaches to addressing communication barriers, and clinical placements in diverse healthcare settings can provide valuable exposure to multicultural patient populations. Structured reflection and debriefing sessions are recommended to help students process these experiences and develop professional competence [16,37].

### 4.5. Implications for Health Policy

Healthcare institutions should develop comprehensive language access policies aligned with international best practices and regulatory requirements, addressing interpreter service provision, multilingual patient materials, staff language competency standards, and cultural responsiveness training. National nursing and healthcare standards should explicitly address cultural competency and communication requirements, providing clear guidance for individual practitioners and institutional policies. Investment in language support infrastructure is essential for ensuring equitable healthcare access for diverse patient populations. Quality indicators related to language access and cultural responsiveness should be incorporated into healthcare accreditation and performance measurement systems, with regular monitoring and reporting to drive continuous improvement in multicultural care delivery [29,36].

### 4.6. Comparison with Existing Literature

The findings align with international research documenting the multifaceted nature of language barriers in healthcare [39,40,41]. However, this study extends existing knowledge by applying ecological systems theory to understand multilevel influences and by exploring the specific context of Saudi healthcare, which serves uniquely diverse patient populations. The identification of informal colleague support as a primary coping strategy resonates with research in other healthcare contexts, while also raising concerns about quality and safety implications of untrained interpretation [42,43,44,45]. The finding that organizational culture significantly influences communication outcomes aligns with research on healthcare quality improvement and patient safety. Unlike previous studies that often focus on single barriers or interventions, this research demonstrates the complex interplay between individual, organizational, and systemic factors, aligning with recent calls for multilevel approaches to addressing healthcare disparities [38].

### 4.7. Strengths and Limitations

Study strengths include the use of a well-established theoretical framework, a diverse sample across multiple healthcare settings, and a comprehensive examination of multilevel factors. Applying ecological systems theory offers a novel lens for understanding communication challenges and identifying targeted interventions. The bilingual data collection (Arabic and English) enhanced the richness and authenticity of responses, while inclusion of both governmental and private facilities provided insights across different organizational contexts.

However, several limitations should be acknowledged. The study focused solely on Saudi nurses’ perspectives, omitting patient experiences and the views of other healthcare professionals involved in communication. Its cross-sectional design limits understanding of how communication strategies evolve over time, and the sample, drawn from four facilities, may restrict transferability to other regional or institutional contexts. Self-selection bias likely influenced participation: nurses who volunteered may have had stronger interest in cross-cultural communication, potentially overrepresenting culturally aware practitioners. The sample’s relatively high proportion with cultural competency training (61.1%) may not reflect the Saudi nursing workforce overall. Future research should use random sampling, incorporate patient perspectives, and employ mixed-methods designs to provide a fuller multilevel account.

### 4.8. Future Research Directions

Future research should incorporate patients’ perspectives on communication challenges and experiences with language support, as understanding both sides of the nurse–patient interaction is essential for developing effective interventions. Comparative studies across different organizational models and support systems could help identify best practices, while longitudinal research is needed to examine how communication competencies evolve over time and in response to specific training or policy interventions.

Intervention studies testing multilevel strategies are particularly important to build evidence for improving cross-cultural communication. Randomized controlled trials evaluating training programs, technological tools, and organizational initiatives could offer practical implementation guidance. Additionally, research on the effectiveness and cost-efficiency of various language support models, such as professional interpreters, digital solutions, and staff training, would inform policy and resource allocation. Mixed-methods approaches integrating qualitative insights with quantitative outcomes (e.g., communication quality, patient satisfaction, and clinical indicators) would strengthen the evidence base for practice and policy development.

## 5. Conclusions

Communication between Saudi nurses and non-Arabic-speaking patients is shaped by intersecting factors across five ecological levels. Language proficiency alone neither predicts nor ensures communication success; cultural competence, peer and institutional support, and policy alignment are equally consequential. The four highest-yield targets for intervention are: cultural competency education integrated throughout nursing curricula; standardized interpreter access protocols specifying when professional interpreters are mandatory; staffing models that account for the additional time cross-language communication requires; and national policy standards with accountability mechanisms for language access equity. The ecological systems framework provides a practical structure for designing and evaluating such coordinated, multilevel interventions.

## Figures and Tables

**Table 1:** Participant Demographics and Professional Characteristics (*n* = 18).

*n* (%)	Characteristic
	Age Range
7 (38.9)	25–30 years
6 (33.3)	31–35 years
3 (16.7)	36–40 years
2 (11.1)	41–45 years
	Education Level
6 (33.3)	Diploma
10 (55.6)	Bachelor’s degree
2 (11.1)	Master’s degree
	Years of Experience
8 (44.4)	1–5 years
6 (33.3)	6–10 years
3 (16.7)	11–15 years
1 (5.6)	16–20 years
	Work Setting
10 (55.6)	Government hospital
8 (44.4)	Private hospital
	Unit/Department
7 (38.9)	Medical–surgical
4 (22.2)	Critical care
3 (16.7)	Emergency department
2 (11.1)	Obstetrics/gynecology
2 (11.1)	Pediatrics
	Self-reported English Proficiency
5 (27.8)	Basic
5 (27.8)	Moderate
8 (44.4)	Advanced
	Cultural Competency Training
11 (61.1)	Yes
7 (38.9)	No
	Weekly Encounters with Non-Arabic Speakers
6 (33.3)	2–5 patients
8 (44.4)	6–10 patients
4 (22.2)	>10 patients

## Data Availability

The qualitative data generated during this study are not publicly available due to privacy and ethical restrictions but may be available from the corresponding author upon reasonable request and subject to ethical approval.

## Supplementary Materials

The following supporting information can be downloaded at: https://www.mdpi.com/article/10.3390/healthcare14142040/s1, Supplementary File S1: COREQ Checklist & Semi-Structured Interview Guide; Supplementary File S2: Semi-Structured Interview Guide.
