# Supplementary material for: Multilevel Factors Influencing Nurse–Patient Communication in Linguistically Diverse Healthcare Settings: A Qualitative Descriptive Study in Saudi Arabia
Source: Healthcare (Basel). 2026 Jul 8;14(14):2040. doi: 10.3390/healthcare14142040 (PMC13409682; doi:10.3390/healthcare14142040)
Supplement: Supplementary file 1 [file healthcare-14-02040-s001.zip › healthcare-4380340-supplementary.pdf]

Supplementary File S1:

COREQ Checklist & Semi-Structured Interview Guide

Consolidated Criteria for Reporting Qualitative Research (COREQ): 32-item checklist

Tong A, Sainsbury P, Craig J. Consolidated criteria for reporting qualitative research (COREQ): a 32-item checklist for interviews and focus groups. Int J Qual Health Care. 2007;19(6):349–357.

No.	Item	Guide questions/description	Reported? (Yes/No/NA)	Section/Page
	Domain 1: Research team and reflexivity			
1	Interviewer/facilitator	Which author/s conducted the interview or focus group?	Yes	§2.4
2	Credentials	What were the researcher's credentials, e.g. PhD, MD?	Yes	§2.4 (bilingual, qualitative research experience stated)
3	Occupation	What was their occupation at the time of the study?	Yes	Author affiliations
4	Gender	Was the researcher male or female?	Yes	§2.7 (team diversity noted; details in reflexivity journals)
5	Experience and training	What experience or training did the researcher have?	Yes	§2.4
6	Relationship established	Was a relationship established prior to study commencement?	Yes	No prior relationship; §2.4
7	Participant knowledge of the interviewer	What did the participants know about the researcher?	Yes	§2.4
8	Interviewer characteristics	What characteristics were reported about the interviewer/facilitator?	Yes	§2.4
	Domain 2: Study design			
9	Methodological orientation and theory	What methodological orientation was stated to underpin the study?	Yes	§2.1, §1.1
10	Sampling	How were participants selected?	Yes	§2.3
11	Method of approach	How were participants approached?	Yes	§2.3
12	Sample size	How many participants were in the study?	Yes	§2.3 (n=18)
13	Non-participation	How many people refused to participate or dropped out?	None, all approached nurses agreed to participate and completed	

			their interview	
14	Setting of data collection	Where was the data collected?	Yes	§2.2, §2.4
15	Presence of non-participants	Was anyone else present besides the participants and researchers?	Yes	§2.4 (one-to-one interviews)
16	Description of sample	What are the important characteristics of the sample?	Yes	§3.1, Table 1
17	Interview guide	Were questions, prompts, guides provided by the authors?	Yes	§2.4; Appendix A
18	Repeat interviews	Were repeat interviews carried out?	No — single interviews per participant; information power guided the final sample size	
19	Audio/visual recording	Did the research use audio or visual recording?	Yes	§2.4
20	Field notes	Were field notes made during and/or after the interview or focus group?	Yes	§2.4
21	Duration	What was the duration of the interviews or focus group?	Yes	§2.4 (45–60 min)
22	Data saturation	Was data saturation discussed?	information power [17] was used instead of saturation	§2.3
23	Transcripts returned	Were transcripts returned to participants for comment and/or correction?	Yes	§2.7 (member-checking)
	Domain 3: Analysis and findings			
24	Number of data coders	How many data coders coded the data?	Yes	§2.6 (two team members coded collaboratively)
25	Description of the coding tree	Did authors provide a description of the coding tree?	Yes	§3.2, Table 2
26	Derivation of themes	Were themes identified in advance or derived from the data?	Yes	§2.6 (inductive coding → deductive mapping)
27	Software	What software, if applicable, was used to manage the data?	No, manual analysis was undertaken	
28	Participant checking	Did participants provide feedback on the findings?	Yes	§2.7 (6 participants checked themes)

29	Quotations presented	Were participant quotations presented to illustrate the themes/findings?	Yes	§3.2 throughout
30	Data and findings consistent	Was there consistency between the data presented and the findings?	Yes	All themes supported by multiple quotations
31	Clarity of major themes	Were major themes clearly presented in the findings?	Yes	§3.2, Table 2
32	Clarity of minor themes	Is there a description of diverse cases or discussion of minor themes?	Yes	§3.3 (Cross-Cutting Patterns)

Supplementary File S2: Semi-Structured Interview Guide

Multilevel Factors Influencing Nurse–Patient Communication with Non-Arabic-Speaking Patients

The following guide was used by bilingual interviewers. Question order was flexible; probes were deployed as needed to elicit depth. The guide was piloted with two nurses not included in the main study.

Section A: Opening and Rapport Building

A1. Can you briefly tell me about your nursing background and your current role?

A2. How frequently do you care for patients who do not speak Arabic?

A3. Which non-Arabic languages do you most commonly encounter?

Section B: Individual-Level Factors (Microsystem)

B1. How would you describe your confidence in communicating with non-Arabic-speaking patients?

B2. Can you describe a specific communication challenge you have experienced with a non-Arabic-speaking patient? What made it difficult?

B3. What communication strategies have you found most effective in these situations?

B4. How has your nursing training prepared you for cross-language communication?

Probes: Do you use non-verbal communication? Visual aids? How do you verify patient understanding?

Section C: Interpersonal and Cultural Factors (Microsystem)

C1. Have you encountered situations where cultural differences—beyond language—affected patient communication? Can you describe one?

C2. How do gender dynamics or family involvement affect your communication with non-Arabic-speaking patients?

C3. How do you identify whether a patient is in pain or distress when you cannot communicate verbally?

Section D: Unit-Level and Organizational Factors (Mesosystem/Exosystem)

D1. What resources does your unit provide to help communicate with non-Arabic-speaking patients?

D2. How does your workload affect your ability to communicate effectively with these patients?

D3. How do your colleagues support you in these situations? Are there multilingual team members?

D4. What interpreter services are available to you? How often do you use them, and how effective are they?

D5. Does your institution have clear protocols for communication with non-Arabic-speaking patients?

Section E: Institutional Policy and System-Level Factors (Macrosystem)

E1. Do you feel your institution's leadership prioritizes effective communication with diverse patient groups? How does this show in daily practice?

E2. How do broader national healthcare policies or professional standards shape your communication practices with non-Arabic-speaking patients?

E3. In your view, what systemic changes would most improve communication in this area?

Section F: Impact and Well-being

F1. How do communication barriers with non-Arabic-speaking patients affect the quality of care you provide?

F2. How do these situations affect your own stress or well-being?

F3. Have you been involved in a situation where a communication barrier contributed to a clinical error or near-miss? (Participants may decline to answer.)

Section G: Closing

G1. Is there anything else you would like to share about your experiences communicating with non-Arabic-speaking patients that we have not covered?

G2. What single change would most improve communication in your setting?
